# A contemporary analysis of disease upstaging of Gleason 3 + 3 prostate cancer patients after robot‐assisted laparoscopic prostatectomy

**DOI:** 10.1002/cam4.6651

**Published:** 2023-11-06

**Authors:** Ruairidh Taggart, Lorenzo Dutto, Hing Y. Leung, Mark Salji, Imran Ahmad

**Affiliations:** ^1^ Glasgow Royal Infirmary Glasgow UK; ^2^ Queen Elizabeth University Hospital Glasgow UK; ^3^ CRUK Scotland Institute The Beatson Institute for Cancer Research Glasgow UK; ^4^ School of Cancer Sciences University of Glasgow Glasgow UK

**Keywords:** active surveillance, Gleason 3+3, prostate cancer, robotic surgery

## Abstract

**Background:**

Risk of biochemical recurrence (BCR) in localised prostate cancer can be stratified using the 5‐tier Cambridge Prognostic Group (CPG) or 3‐tier European Association of Urology (EAU) model. Active surveillance is the current recommendation if CPG1 or EAU low‐risk criteria are met. We aimed to determine the contemporary rates of upgrading, upstaging and BCR after radical prostatectomy for CPG1 or EAU low‐risk disease.

**Methods:**

A database of all robotic‐assisted laparoscopic prostatectomies (RALPs) performed in Glasgow between 12/2015 and 05/2022 was analysed. Rates of upgrading, upstaging and BCR post‐RALP for CPG1 or EAU low‐risk disease were defined. Univariate and multivariate analysis were performed to assess the relationship between patient factors and outcomes.

**Results:**

A total of 1223 RALP cases were identified. A total of 12.6% met CPG1 criteria with 70.1% and 25.3% upgraded and upstaged to extraprostatic disease post‐operatively respectively. A total of 5.8% met EAU low‐risk criteria with 60.6% upgraded and 25.4% upstaged to extraprostatic disease post‐operatively respectively. CPG1 (*p* < 0.0001) and EAU low‐risk (*p* = 0.02) patients were at a significantly higher risk of BCR if upstaged.

**Discussion:**

Many patients who met CPG1 or EAU low‐risk criteria were upgraded post‐RALP and approximately 25% were upstaged due to extraprostatic disease. Upstaging puts patients at a significantly higher risk of BCR.

## 1 BACKGROUND

Prostate cancer (PC) is the most common cancer in males in the United Kingdom and each year 1.3 million men are diagnosed worldwide.^1^, ^2^ The introduction of ad hoc PSA screening as well as a greater availability of MRI scanners has meant that PC is now being diagnosed at a less advanced stage.^3^ The PROMIS study demonstrated that using multiparametric MRI (mpMRI) to triage patients prior to biopsy, both improves the detection of clinically significant PC and reduces over diagnosis of clinically insignificant PC.^4^ However, despite these advances, there remains a significant morbidity and reduced quality of life associated with over diagnosis and over treatment.^2^ Although the introduction of robotic‐assisted laparoscopic prostatectomy (RALP) has reduced complication rates and improved outcomes, issues such as erectile dysfunction and urinary incontinence still persist.^5^, ^6^ The ProtecT study found that 85% and 20% of patients reported persisting erectile dysfunction and incontinence, respectively, 6 years post‐prostatectomy.^7^ Currently, biopsy Gleason grade, clinical T stage and PSA level are prognostic indicators used in combination to stratify patient risk in order to better inform them when deciding whether to proceed with surgical management for PC.

The Gleason grading system is an established prognostic tool in PC and Gleason 3 + 3 = 6 is currently the lowest grade available, reflecting it being the most well‐differentiated tumour pattern.^3^, ^8^ There has been growing pressure to reclassify Gleason 3 + 3 = 6 as a benign entity given the evidence that few men die from this disease.^9^ Rightly, many urologists now advocate an active surveillance (AS) approach in these patients as opposed to radical treatment due to treatment‐related morbidity concerns.^10^ Klotz et al. demonstrated that the mortality rate of those managed with AS is consistent with that of patients who had an initial definitive intervention over a 15‐year period.^11^, ^12^, ^13^ As a result, there has been a greater focus on identifying and incorporating reliable prognostic indicators in patients diagnosed with low‐grade disease.

The National Institute for Health and Care Excellence recommends that the 5‐tier Cambridge Prognostic Group (CPG) model be used to stratify risk of biochemical recurrence (BCR) in localised or locally advanced PC.^14^ Previous guidance had used a 3‐tier model still used by the European Association of Urology (EAU) which divides patients into low, intermediate and high risk for BCR.^15^ Evidence suggests that the CPG model provides a more reliable prediction of PC‐specific mortality and is therefore of greater use when counselling patients.^14^ Both models utilise biopsy‐reported Gleason grade, T stage on digital rectal examination and pre‐operative PSA level (Table 1).^14^, ^15^ CPG 1 reflects a Gleason grade of 3 + 3 = 6, clinical T stage 1 or 2 and PSA <10 ng/mL.^14^ Whereas low‐risk disease, using the EAU model, differs in that stage is specified as being ≤T2a.^15^ AS is the current recommendation if either of these criteria are met.^14^, ^15^

**TABLE 1:** CPG 1 versus EAU low‐risk disease criteria.

	CPG 1	EAU low risk
Biopsy Gleason grade	3 + 3 = 6	3 + 3 = 6
Clinical T stage	<T3a	≤T2a
PSA (ng/mL)	<10	<10

Abbreviations: CPG, Cambridge Prognostic Group; EAU, European Association of Urology.

The objectives of our study were to determine the contemporary rates of upgrading, upstaging and BCR in patients who have a radical prostatectomy for CPG 1 or EAU low‐risk disease, and whether this has implications for future counselling of patients considering AS.

## 2 METHODS

### 2.1 Patient selection and study design

A database of all RALPs performed at the Queen Elizabeth University Hospital in Glasgow between December 2015 and May 2022 was analysed retrospectively. Pre‐operative Gleason grades on biopsies, T stages on MRI and PSA levels (ng/mL) were recorded. Patient age at the time of RALP, date of biopsy, any presence of perineural or lymphovascular invasion on biopsy and date of MRI were also documented. Clinical T stages were replaced by radiological T stages for use in risk stratification models. Patients who met the criteria for CPG 1 (Gleason 3 + 3 on biopsy, T stage <3a and PSA level <10 ng/mL) were selected for further analysis. These patients were also subcategorised into those who the met criteria for EAU low‐risk PC (Gleason 3 + 3 on biopsy, T stage ≤2a and PSA level <10 ng/mL) to demonstrate any significant prognostic differences between the two.

Prostatectomy pathology reports were reviewed, and their definitive Gleason grades and T stages were recorded to assess the rate of upgrading and upstaging respectively. Referring health board, prostatectomy specimen weight, any presence of perineural or lymphovascular invasion and margin status were also recorded.

Post‐operative PSA levels were recorded and followed up until March 2023 to determine the risk of BCR in the CPG 1 and EAU low‐risk cohorts. Multivariate analysis was performed to identify factors that could predict BCR.

### 2.2 Statistical analysis

Mean, median, range and standard deviations were generated for continuous variables, and frequencies and proportions were generated for categorical variables. All data analysis was performed using GraphPad Prism Version 9. All P values were two‐sided, with the significance level defined as *p* < 0.05.

Multivariate analysis using Cox's proportional hazards (PH) models were performed in R (R version 4.0.4 package ‘survival’) with significance taken as *p* < 0.05 and indicated by *. All pre‐ and post‐operative factors with relevance to BCR were included in the models which were separately generated for CPG 1 and EAU low‐risk cohorts. The PH assumption was checked for each cohort using package ‘survminer’, with no co‐variates scaled Schoenfeld residuals significantly correlating with time supporting the assumption of PH.

## 3 RESULTS

The database consisted of 1223 RALP cases. 154/1223 patients (12.6%) met the criteria for CPG 1 and had an average age of 62. The average pre‐operative PSA level across the cohort was 6.6 ng/mL. The average percentage of biopsy cores positive for Gleason 3 + 3 disease was 37%. Perineural invasion was present in 18.2% of biopsy cores. The average prostatectomy specimen weight was 44.1 g. 121/154 patients (78.6%) had perineural invasion present in their prostatectomy specimen. Only one prostatectomy specimen had evidence of lymphovascular invasion. 40/154 patients (26%) were found to have a positive margin in their prostatectomy specimen (Table 2).

**TABLE 2:** Comparison of patient characteristics, pre‐operative and post‐operative prognostic indicators in CPG1 and EAU low‐risk cohorts.

	CPG 1	EAU low risk
*Patient characteristics*		
Age (years)	*n* = 154^a^	*n* = 71^a^
Average	62	61.8
Median	62.2	62.7
Standard deviation	6.8	6.6
Range	43.6–76.3	43.6–75.3
*Pre‐operative prognostic indicators*		
PSA (ng/mL)	*n* = 154^a^	*n* = 71^a^
Average	6.6	6.5
Median	6.4	6.1
Standard deviation	1.9	1.9
Range	0.2–9.9	0.2–9.9
% positive biopsy cores	*n* = 147^a^	*n* = 68^a^
Average	37	28.8
Median	31.3	25
Standard deviation	24.7	21.8
Range	5.9–100	5.9–100
Perineural invasion (biopsy)	*n* = 148^a^	*n* = 69^a^
Yes (%)	27 (18.2)	6 (8.7)
No (%)	121 (81.8)	63 (91.3)
*Post‐operative prognostic indicators*		
Prostate weight (g)	*n* = 148^a^	*n* = 66^a^
Average	44.1	46.7
Median	40	42
Standard deviation	16.8	20.6
Range	16–121	16–121
Perineural invasion (prostatectomy)	*n* = 154^a^	*n* = 71^a^
Yes (%)	121 (78.6)	47 (66.2)
No (%)	33 (21.4)	24 (33.8)
Lymphovascular invasion (prostatectomy)	*n* = 154^a^	*n* = 71^a^
Yes (%)	1 (0.6)	0 (0)
No (%)	153 (99.4)	71 (100)
Positive margin	*n* = 154^a^	*n* = 71^a^
Yes (%)	40 (26)	17 (23.9)
No (%)	114 (74)	54 (76.1)

Abbreviations: CPG, Cambridge Prognostic Group; EAU, European Association of Urology.

^a^ *n* variable due to unavailable patient data.

**FIGURE 1:**
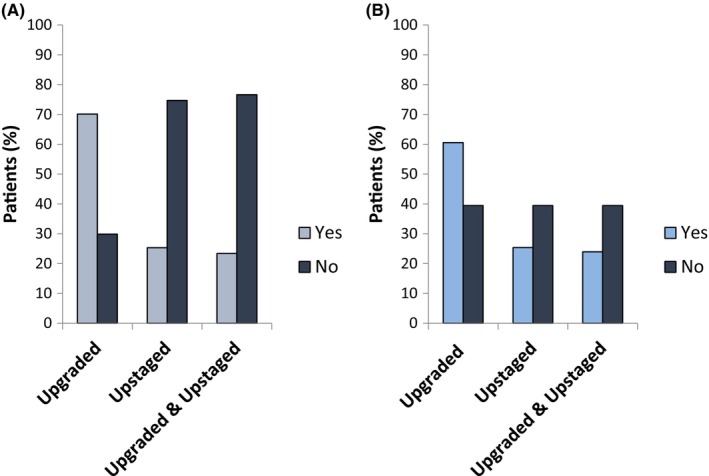
Post‐operative Gleason upgrading and T upstaging in patients who met (A) CPG 1 (*n* = 154) and (B) EAU low‐risk (*n* = 71) criteria pre‐operatively. CPG, Cambridge Prognostic Group; EAU, European Association of Urology.

108/154 patients (70.1%) who met CPG 1 criteria pre‐operatively had their Gleason upgraded on the final prostatectomy pathology report compared to their pre‐operative biopsy. 39/154 patients (25.3%) were upstaged from their radiological T stage and were found to have pathological stage T3 disease post‐operatively. 36/154 patients (23.4%) were Gleason upgraded and T upstaged post‐operatively (Figure 1). Dates of biopsies and MRI were available for 38 of the patients who were upstaged, and 22/38 patients (57.9%) had been staged prior to biopsy (Table S1). Rate of upgrading ranged from 64.7% to 83.3% between referring health boards, whereas upstaging ranged from 11.1% to 45.8% in the CPG 1 cohort (Table S2). 13/39 patients (33.3%) who were upstaged had initially been enrolled on AS and had transitioned to an active treatment plan.

**FIGURE 2:**
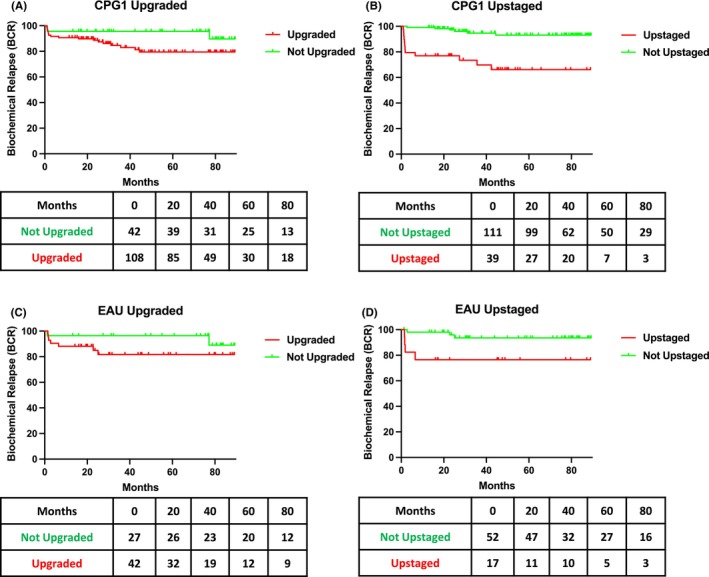
Biochemical recurrence risk in (A) CPG 1 upgraded, (B) CPG 1 upstaged, (C) EAU low‐risk upgraded and (D) EAU low‐risk upstaged. *n = 150 in CPG 1 cohort and *n* = 69 in EAU low‐risk cohort. CPG, Cambridge Prognostic Group; EAU, European Association of Urology.

Post‐operative PSA levels were available for 150/154 CPG 1 patients (97.4%). 21/150 patients (14%) developed BCR over the follow‐up period. The average follow‐up period was 45.5 months. Patients who met CPG 1 criteria and had their Gleason upgraded post‐operatively were not at significantly higher risk of BCR, with 18/108 patients (16.7%) identified. Whereas CPG 1 patients who were T upstaged post‐operatively were at a statistically significant higher risk of developing BCR, with 12/39 patients (30.8%) identified (Figure 2). 11/18 CPG 1 patients (61.1%) who developed BCR after being Gleason upgraded had a positive margin compared to 9/12 patients (75%) who had been upstaged. Our multivariate model identified upstaging and positive margin as independent predictors of BCR, after controlling for other factors, in patients who met CPG 1 criteria pre‐operatively (Table 3).

**TABLE 3:** Multivariate analysis of CPG 1 cohort including possible factors which could influence time to BCR.

	Hazard ratio	Lower confidence interval	Upper confidence interval	*p‐*Value
Upgraded	1.64	0.45	6.02	0.452
Upstaged	2.97	1.08	8.15	0.034 *
Positive margin	3.11	1.12	8.61	0.029*
Pre‐op major T stage (1, 2)	1.9	0.52	6.97	0.334
Pre‐op PSA ≥6 ng/mL	0.79	0.32	1.96	0.618
Biopsy cores ≥6	0.63	0.24	1.66	0.354
Perineural invasion in prostatectomy specimen	1.08	0.2	5.75	0.93

*Note*: *n* = 148, number of events = 20 (sufficient data not available for six patients). ^*^ *p* < 0.05.

Abbreviations: BCR, biochemical recurrence; CPG, Cambridge Prognostic Group.

71/1223 patients (5.8%) met the criteria for EAU low‐risk disease and had an average age of 61.8 years. The average pre‐operative PSA level across the cohort was 6.5 ng/mL. The average percentage of biopsy cores positive for Gleason 3 + 3 disease was 28.8%. Perineural invasion was present in 8.7% of core biopsies. The average prostatectomy specimen weight was 46.7 g. 47/71 patients (66.2%) had perineural invasion present in their prostatectomy specimen. No prostatectomy specimen had evidence of lymphovascular invasion. 17/71 patients (23.9%) were found to have a positive margin in their prostatectomy specimen (Table 2).

43/71 patients (60.6%) who met EAU low‐risk criteria pre‐operatively had their Gleason upgraded on the final prostatectomy pathology report compared to their pre‐operative biopsy. 18/71 patients (25.4%) were upstaged from their radiological T stage and were found to have pathological stage T3 disease post‐operatively. 17/71 patients (23.9%) were Gleason upgraded and T upstaged post‐operatively (Figure 1). Dates of biopsies and MRI were available for all 18 of the patients who were upstaged, and 12/18 patients (66.7%) had been staged prior to biopsy (Table S1). Rate of upgrading ranged from 0% to 75% between referring health boards, whereas upstaging ranged from 0% to 50% in the EAU low‐risk cohort (Table S3). 6/18 patients (33.3%) who were upstaged had initially been enrolled on AS and had transitioned to an active treatment plan.

Post‐operative PSA levels were available for 69/71 EAU low‐risk patients (97.2%). 9/69 patients (13%) developed BCR over the follow‐up period. The average follow‐up period was 50.1 months. Patients who met EAU low‐risk criteria and had their Gleason upgraded post‐operatively were not at significantly higher risk of BCR, with 7/43 patients (16.3%) identified. Whereas EAU low‐risk patients who were T upstaged post‐operatively were at a statistically significant higher risk of developing BCR, with 4/18 patients (22.2%) identified (Figure 2). 4/7 EAU low‐risk patients (57.1%) who developed BCR after being Gleason upgraded had a positive margin compared to 3/4 patients (75%) who had been upstaged. Our multivariate model identified positive margin as the only independent predictor of BCR, after controlling for other factors, in patients who met EAU low‐risk disease criteria pre‐operatively (Table 4).

**TABLE 4:** Multivariate analysis of EAU low‐risk cohort including possible factors which could influence time to BCR.

	Hazard ratio	Lower confidence interval	Upper confidence interval	*p*‐Value
Upgraded	2.28	0.4	12.79	0.34
Upstaged	0.33	0.04	2.48	0.28
Positive margin	9.34	1.25	69.6	0.029*
Pre‐op major T stage (1, 2)	2.01	0.44	9.07	0.36
Pre‐op PSA ≥6 ng/mL	0.29	0.05	1.43	0.13
Biopsy cores ≥6	0.44	0.05	3.87	0.46
Perineural invasion in prostatectomy specimen	4.24	0.44	40.7	0.21

*Note*: *n* = 68, number of events = 9 (sufficient data not available for three patients). ^*^ *p* < 0.05.

Abbreviations: BCR, biochemical recurrence; EAU, European Association of Urology.

## 4 DISCUSSION

While AS is the current recommendation, 12.6% and 5.8% of our patients had undergone a RALP for CPG 1 and EAU low‐risk disease respectively. The support for AS centres around the aim of reducing patient morbidity from unnecessary active treatment.^10^, ^16^ Hamdy et al. have previously shown that a period of AS does not have a significant impact on mortality in this who subsequently switch to active treatment.^17^, ^18^ While this can help reassure patients, often anxiety surrounding the fact that they are living with untreated ‘cancer’ plays a role in the decision to choose active treatment and this has likely been reflected in our database.^16^ In both the CPG1 and EAU low‐risk cohorts, 33.3% of patients had previously been enrolled on AS. Radiological disease progression on MRI and/or rising PSA were the reasons given for transitioning to active treatment in all but one patient who had progressive, bothersome lower urinary tract symptoms.

The majority of patients with CPG 1 and EAU low‐risk disease were upgraded to clinically significant disease (Gleason ≥7) post‐operatively, at 70.1% and 60.6% respectively. The difference in Gleason upgrading between the two risk models we used can likely be attributed to the cohort size more than doubling with the CPG model due to the inclusion of T2b/c disease. This in theory equates to a more advanced and poorly differentiated disease, increasing the likelihood of failure to capture a representative field on biopsy.^19^ Capturing the highest Gleason grade of the tumour with a core biopsy remains challenging; however, pre‐biopsy MRI and fusion biopsy techniques have improved the diagnosis of clinically significant disease.^4^, ^20^ Lacetera et al. found that using an MRI/US fusion biopsy technique more than doubled the detection of clinically significant disease in men on AS.^21^ We defined a post‐operative Gleason grade of ≥7 as being clinically significant as this would have resulted in patients being classified as having a higher risk disease pre‐operatively using both the CPG and EAU models. AS is a well‐established management option in the context of CPG 1 and EAU low‐risk disease.^15^ While patients with Gleason 7 disease on biopsy can be candidates for AS, stricter criteria need to be met and the evidence in support of this option is limited.^15^ Classifying upgrading as Gleason ≥7 therefore increases the utility of our results in counselling patients with CPG 1 or EAU low‐risk disease on AS compared to surgical management.

Rates of upstaging to T3 disease were similar when using the CPG 1 or EAU low risk criteria, at approximately 25%. We did not classify a change in T2 subtype as a significant upstaging. This view is derived from that of the Union for International Cancer Control who now no longer recognise pathological T2 subtypes.^15^ The Royal College of Pathologists' data set for reporting prostate carcinomas states that subtyping T2 disease is unlikely to offer any significant prognostic benefit.^22^ They highlight that a small midline tumour (T2c disease) is unlikely to be consistently more aggressive than a larger unilateral tumour.^22^ This same concern can be levelled at subtyping T2 disease clinically and radiologically. The EAU currently classifies patients with clinical T stage 2b and 2c disease as intermediate and high risk, respectively, and would recommend radical prostatectomy be considered.^15^ The Royal College of Pathologists has reservations about whether true T2b disease is actually feasible. They argue that a tumour large enough to fill over half a lobe is likely to have already crossed the midline and/or extend out with the prostate capsule.^22^ Our results have further supported the argument that subtyping stage T2 disease is of little prognostic value and should not be a primary driver in treatment decisions as rates of upstaging to extraprostatic disease were similar in both the CPG 1 and EAU low‐risk cohorts.

Patients who meet CPG 1 or EAU low‐risk criteria may understandably be hesitant about choosing AS given that we have demonstrated a 25% chance of having extraprostatic disease. We have also shown that upstaging puts patients at a significantly higher risk of BCR, whereas upgrading does not appear to be influential. These results support an extensive disease being more operatively challenging to clear compared to a smaller more poorly differentiated tumour. BCR rate was similar in both the CPG 1 and EAU low‐risk cohort meaning that T2 subtype does not appear to be a pivotal factor. This raises further doubts about the EAU model as T2b and T2c would equate to intermediate and high risk respectively.^15^ Our multivariate analysis reiterated the association between upstaging and BCR in the CPG 1 cohort, but interestingly failed to show significance in EAU low‐risk disease. We believe that this inconsistency can be attributed to a smaller sample size in the EAU low‐risk cohort. Martini et al. demonstrated that a positive margin is associated with an increased risk of BCR; however, our results have also shown that margin status can be used as an independent predictor of BCR when other factors are controlled in patients with CPG 1 and EAU low‐risk disease pre‐operatively.^23^ Surgeon experience was not included in our multivariate analysis; however, previous work by Bravi et al. reported no statistically significant relationship between surgeon experience and BCR rates post‐RALP.^24^ Previous studies have already demonstrated that the mortality rate in those receiving AS versus those receiving radical treatment is similar.^11^ It should be stressed that we did not investigate PC‐specific mortality and our results on upstaging and BCR should not be interpreted as being associated with this. Our results will enable surgeons to provide stronger evidence‐based counselling to patients when deciding between AS and RALP in the context of CPG 1 and EAU low‐risk disease and also help refine our post‐operative protocols.

We have demonstrated the utility of using radiological as opposed to clinical staging in risk stratification models. Park et al. showed that patients with Gleason 3 + 3 disease and a visible tumour on MRI have a 49.8% chance of being upgraded post‐prostatectomy.^25^, ^26^ Furthermore, MRI can be used like ultrasound to measure prostate size and previous studies have suggested an inverse relationship between size and Gleason upgrading.^25^, ^27^, ^28^ We have shown that integrating MRI results into prognostic calculations provides a more accurate and reliable impression of the disease.

It is now standard practice to stage patients radiologically using MRI prior to obtaining biopsies, as concerns had been raised about post‐biopsy changes influencing how images are interpreted. Our study included cases over several years, and we therefore wanted to determine whether timing of MRI might have impacted our rates of upstaging. We found that most patients had been radiologically staged prior to biopsy regardless of whether they were upstaged in both the CPG 1 and EAU low‐risk cohorts.

There have been a number of studies which have reported the presence of cribriform glands and intraductal carcinoma of the prostate (IDC‐P) being associated with more aggressive PC and poorer prognosis.^29^ There has been debate between pathologists as to whether the presence of cribriform glands should equate to Gleason pattern 3 or 4. The current recommendation from the Royal College of Pathologists is that their presence should indicate Gleason pattern 4.^22^ Explicit reporting of the presence or absence of cribriform glands is currently not included as a core data set item for the histopathological reporting of PC.^22^ Therefore, a proportion of reported Gleason 4 in our cohort will include cribriform glands but it is not possible to know which. The explicit reporting of IDC‐P is also not included as a core data set item for pathology reports.^22^ However, a small proportion of the prostate biopsy reports reviewed for this cohort did specifically mention the presence or absence of IDC‐P. In all of these cases, IDC‐P was absent.

Limitations of this study include the fact that it was not a randomised control trial. We did not include cases managed with radical radiotherapy, so selection bias may have influenced our rates of upgrading and upstaging after active treatment. There may have been variation in how MRI sequences were performed, and images were interpreted between different hospitals. Patients referred from Unit 2 had proportionally higher rates of upstaging and we hypothesise that this is secondary to the lack of a dedicated uroradiologist in that health board. There are also variations in biopsy protocols and, despite attempts to help standardise the process, those taking the biopsies use different techniques. Due to the documentation available, we were unable to assess if the level of experience of the individual performing the biopsy was influential on upgrading rates. Interobserver variation between reporting pathologists may have also influenced our results given that this was a retrospective study, and we were therefore unable to assess agreeability.

## 5 CONCLUSIONS

Our study has demonstrated in our ‘real‐world’ cohort, that most patients who meet the criteria for CPG 1 or EAU low risk disease pre‐operatively are subsequently upgraded post‐RALP and approximately 25% are upstaged to extraprostatic disease. Upstaging puts patients at a significantly higher risk of BCR. We will be able to use this data to better inform our patients when counselling them on AS.

## AUTHOR CONTRIBUTIONS

**Ruairidh Taggart:** Formal analysis (equal); methodology (equal); writing – original draft (equal); writing – review and editing (equal). **Lorenzo Dutto:** Writing – review and editing (supporting). **Hing Y. Leung:** Writing – review and editing (supporting). **Mark Salji:** Formal analysis (equal); writing – original draft (supporting); writing – review and editing (supporting). **Imran Ahmad:** Conceptualization (lead); writing – original draft (lead); writing – review and editing (lead).

## FUNDING INFORMATION

This work was supported by Cancer Research UK Clinician Scientist Fellowship award to Dr. Imran Ahmad (C49745/A19661).

## CONFLICT OF INTEREST STATEMENT

No potential conflicts of interest.

## ETHICS STATEMENT

This study was approved by the Local NHS Ethical Committee, with prior written informed consent obtained from study participants.

## Supporting information


Table S1:

## Data Availability

NA.
