# Supplementary material for: A contemporary analysis of disease upstaging of Gleason 3 + 3 prostate cancer patients after robot‐assisted laparoscopic prostatectomy
Source: Cancer Med. 2023 Nov 6;12(22):20830–7. doi: 10.1002/cam4.6651 (PMC10709727; doi:10.1002/cam4.6651)
Supplement: Supplementary file 1 — Table S1: [file CAM4-12-20830-s001.docx]

**Supplementary Tables**

**Supplementary Table 1:** Comparison of patients who had radiological staging pre- vs. post-biopsy. *n variable due to unavailable patient data.

|  | **CPG 1** | | **EAU Low Risk** | |
| --- | --- | --- | --- | --- |
|  | **Upstaged**  **(n=38)** | **Not upstaged (n=108)** | **Upstaged (n=18)** | **Not**  **upstaged**  **(n=49)** |
| **MRI prior to biopsy (%)** | 22  (57.9) | 72  (66.7) | 12  (66.7) | 28  (57.1) |
| **Biopsy prior to MRI (%)** | 16  (42.1) | 36  (33.3) | 6  (33.3) | 21  (42.9) |

**Supplementary Table 2:** Breakdown of upgrading and upstaging rates between referring health boards for patients who met CPG 1 criteria pre-operatively.

| **Region** | **Upgraded (%)** | **Upstaged (%)** | **Both (%)** |
| --- | --- | --- | --- |
| Unit 1 (n=92) | 66.3 | 19.6 | 18.5 |
| Unit 2 (n=24) | 83.3 | 45.8 | 41.7 |
| Unit 3 (n=17) | 64.7 | 35.3 | 29.4 |
| Unit 4 (n=12) | 83.3 | 25.0 | 25.0 |
| Unit 5 (n=9) | 66.7 | 11.1 | 11.1 |

**Supplementary Table 3:** Breakdown of upgrading and upstaging rates between referring health boards for patients who met EAU low risk criteria pre-operatively.

| **Region** | **Upgraded (%)** | **Upstaged (%)** | **Both (%)** |
| --- | --- | --- | --- |
| Unit 1 (n=46) | 58.7 | 21.7 | 19.6 |
| Unit 2 (n=8) | 75.0 | 50.0 | 50.0 |
| Unit 3 (n=10) | 70.0 | 30.0 | 30.0 |
| Unit 4 (n=4) | 75.0 | 25.0 | 25.0 |
| Unit 5 (n=3) | 0 | 0 | 0 |
